# Supplementary material for: A Cohort Study of the Influence of the 12-Component Modified Japanese Diet Index on Oral and Gut Microbiota in the Japanese General Population
Source: Nutrients. 2024 Feb 13;16(4):524. doi: 10.3390/nu16040524 (PMC10893011; doi:10.3390/nu16040524)
Supplement: Supplementary file 1 [file nutrients-16-00524-s001.zip › nutrients-2845474-supplementary.pdf]

Supplementary Table S1. The amount of change in foods and food groups intake from 2018 to 2018.

	L_1-L_2 (n = 156)	H_1-L_2 (n = 65)	L_1-H_2 (n = 42)	H_1-H_2 (n = 133)	p-value
Soybeans and soybean foods	0.5 (-6.8-8.6)	-3.1 (-12.8-4.3)	5.2 (-5.0-15.1)	4.2 (-8.9-18.0)	0.014
Green and yellow vegetables	-0.4 (-9.3-9.3)	-5.5 (-17.6-3.3)	1.3 (-10.6-18.2)	-1.4 (-17.6-15.4)	0.061
Fruit	0.0 (-6.6-7.9)	-3.0 (-13.6-6.5)	3.0 (-5.8-16.4)	0.4 (-11.8-11.2)	0.275
Fish and shellfish	-0.9 (-8.4-12.7)	-2.8 (-16.4-6.0)	7.7 (-11.5-17.6)	2.1 (-10.6-19.7)	0.105
Pickles	0.0 (-1.2-1.0)	-0.2 (-2.8-0.1)	0.1 (-1.6-1.4)	0.0 (-2.5-1.5)	0.208
Mushrooms	0.1 (-1.2-1.4)	-1.3 (-4.2-0.3)	0.6 (-0.5-2.6)	0.2 (-1.7-2.9)	<0.001
Seaweeds	0.0 (-1.8-1.5)	-2.1 (-5.3-0.5)	0.6 (-1.0-6.5)	0.0 (-3.7-3.4)	<0.001
Green tea	0.0 (-14.9-12.4)	0.0 (-21.3-24.6)	1.8 (-4.5-37.9)	-1.4 (-31.5-36.4)	0.271
Rice	-4.3 (-46.0-25.1)	-9.1 (-39.5-17.8)	0.0 (-22.3-25.2)	-3.4 (-28.2-26.7)	0.658
Miso soup	0.0 (-24.5-26.5)	-5.6 (-38.6-14.5)	0.1 (-20.7-15.6)	-3.5 (-26.0-23.7)	0.408
Beef and pork	0.0 (-4.5-5.6)	1.0 (-2.5-10.4)	-0.3 (-9.3-2.2)	0.0 (-2.7-5.2)	0.101
Coffee	2.9 (-22.1-31.8)	0.4 (-17.1-50.0)	0.7 (-13.6-17.4)	-0.3 (-19.7-14.7)	0.464

Median (range). L_1-L_2: mJDI12 ≤ 6 in 2017 and 2018; H_1-L_2: mJDI12 ≥ 7 in 2017 and ≤6 in 2018; L_1-H_2: mJDI12 ≤ 6 in 2017 and mJDI12 ≥ 7 in 2018; H_1-H_2: mJDI12 ≥ 7 in 2017 and 2018.

Supplementary Table S2. Correlation of the amount of the change for one year between food and bacteria abundance (L_1-L_2).

		Soybeans and soybean foods	Green/yellow vegetables	Fruit	Fish/shellfish	Pickles	Mushrooms	Seaweeds	Green tea	Rice	Miso soup	Beef and pork	Coffee
Oral	Genus												
	Alloprevotella	-0.070	0.096	-0.135	-0.045	-0.067	0.085	0.141	0.107	0.083	0.082	-0.028	-0.013
Gut	Phylum												
	Firmicutes	0.052	-0.103	0.065	-0.138	0.074	0.145	-0.010	-0.087	0.099	-0.030	0.019	-0.017
	Actinobacteria	-0.041	-0.017	-0.112	0.012	0.015	-0.031	0.066	0.150	0.038	0.013	-0.136	-0.078
	Class												
	Clostridia	0.066	-0.057	0.026	-0.136	0.082	0.169*	0.014	-0.155	0.033	-0.007	0.092	-0.040
	Negativicutes	-0.159*	0.098	-0.050	0.078	-0.120	-0.001	-0.033	0.147	0.056	0.110	-0.059	0.134
	Actinobacteria	-0.041	-0.017	-0.112	0.012	0.015	-0.031	0.066	0.150	0.038	0.013	-0.136	-0.078
	Order												
	Clostridiales	0.066	-0.057	0.026	-0.137	0.082	0.169*	0.014	-0.155	0.034	-0.007	0.091	-0.039
	Selenomonadales	-0.159*	0.098	-0.050	0.078	-0.120	-0.001	-0.033	0.147	0.056	0.110	-0.059	0.134
	Bifidobacteriales	-0.046	-0.045	-0.081	0.023	-0.053	-0.016	0.077	0.182*	0.045	0.034	-0.149	-0.052
	Family												
	Ruminococcaceae	0.058	0.001	-0.014	-0.145	0.030	0.027	-0.023	-0.089	0.044	-0.178*	-0.141	-0.101
	Veillonellaceae	-0.169*	0.023	-0.052	0.088	-0.142	-0.065	-0.058	0.170*	0.059	0.107	-0.026	0.071
	Bifidobacteriaceae	-0.046	-0.045	-0.081	0.023	-0.053	-0.016	0.077	0.182*	0.045	0.034	-0.149	-0.052
	Actinomycetaceae	-0.116	-0.098	-0.100	0.075	0.135	-0.063	-0.068	0.023	-0.063	-0.044	0.030	-0.013
	Genus												
	Faecalibacterium	0.010	-0.032	0.053	-0.109	0.055	-0.013	0.003	0.146	0.052	0.046	-0.041	-0.019
	Gemmiger	0.093	0.135	-0.010	-0.063	0.056	0.045	0.023	-0.030	-0.077	0.001	0.050	-0.048
	Ruminococcus	0.104	0.075	0.045	-0.035	-0.077	0.047	-0.006	-0.143	0.009	-0.152	-0.103	-0.057
	Lachnospiracea_incertae_sedis	-0.205*	-0.197*	0.006	0.072	-0.044	-0.047	-0.022	-0.011	-0.100	0.061	0.082	0.090
	Bifidobacterium	-0.047	-0.044	-0.082	0.023	-0.054	-0.016	0.077	0.181*	0.045	0.033	-0.151	-0.053
	Actinomyces	-0.129	-0.094	-0.095	0.076	0.134	-0.059	-0.064	0.041	-0.063	-0.042	0.033	-0.017
	Parabacteroides	-0.030	0.063	0.009	0.037	0.044	-0.030	-0.088	-0.053	-0.022	-0.107	-0.011	0.162*

Spearman's rank correlation coefficient.

*: q-value(False Discovery Rate (Benjamini & Hochberg)) < 0.05

Supplementary Table S3. Correlation of the amount of the change for one year between food and bacteria abundance (H_1-L_2).

		Soybeans and soybean foods	Green/yellow vegetables	Fruit	Fish/shellfish	Pickles	Mushrooms	Seaweeds	Green tea	Rice	Miso soup	Beef and pork	Coffee
Oral	Genus												
	Alloprevotella	-0.093	-0.092	-0.037	-0.030	0.039	0.122	-0.073	0.145	-0.285*	0.085	-0.055	-0.025
Gut	Phylum												
	Firmicutes	0.114	-0.061	0.142	0.050	0.135	0.013	0.199	-0.201	0.044	-0.198	0.151	-0.027
	Actinobacteria	-0.036	-0.110	0.094	-0.076	-0.130	-0.249*	-0.265*	0.087	-0.124	0.018	-0.202	-0.193
	Class												
	Clostridia	0.213	0.025	0.173	-0.003	0.123	-0.017	0.112	-0.160	0.043	-0.144	0.119	-0.092
	Negativicutes	-0.202	0.156	-0.191	-0.062	-0.025	0.024	0.070	-0.212	-0.017	-0.029	-0.033	0.063
	Actinobacteria	-0.036	-0.110	0.094	-0.076	-0.130	-.249*	-.265*	0.087	-0.124	0.018	-0.202	-0.193
	Order												
	Clostridiales	0.213	0.024	0.172	-0.004	0.122	-0.017	0.113	-0.160	0.044	-0.144	0.119	-0.091
	Selenomonadales	-0.202	0.156	-0.191	-0.062	-0.025	0.024	0.070	-0.212	-0.017	-0.029	-0.033	0.063
	Bifidobacteriales	-0.067	-0.072	0.083	-0.021	-0.074	-0.110	-0.147	0.129	-0.139	0.053	-0.215	-0.146
	Family												
	Ruminococcaceae	0.082	0.048	0.148	-0.030	-0.099	-0.035	-0.039	0.070	-0.040	0.203	0.105	-0.230
	Veillonellaceae	-0.049	0.000	-0.144	0.028	-0.059	-0.218	-0.153	-0.242	-0.106	-0.068	-0.058	0.072
	Bifidobacteriaceae	-0.067	-0.072	0.083	-0.021	-0.074	-0.110	-0.147	0.129	-0.139	0.053	-0.215	-0.146
	Actinomycetaceae	-0.021	-0.017	0.071	-0.135	0.148	-0.030	0.124	0.033	0.032	0.115	0.147	-0.005
	Genus												
	Faecalibacterium	0.087	0.079	0.040	-0.026	0.115	0.030	0.153	-0.149	-0.054	0.157	-0.026	0.079
	Gemmiger	0.153	-0.152	-0.025	0.032	0.153	-0.046	-0.126	-0.126	0.094	0.274*	0.094	-0.199
	Ruminococcus	-0.018	0.103	-0.038	0.100	-0.241	0.065	0.036	0.095	-0.111	0.058	0.078	-0.157
	Lachnospiracea_incertae_sedis	-0.013	0.203	0.093	0.199	0.242	-0.013	-0.260*	-0.186	-0.122	0.127	-0.115	0.020
	Bifidobacterium	-0.067	-0.072	0.083	-0.021	-0.074	-0.110	-0.147	0.129	-0.139	0.053	-0.215	-0.146
	Actinomyces	-0.028	0.000	0.070	-0.119	0.147	-0.027	0.145	0.042	0.031	0.129	0.142	-0.001
	Parabacteroides	-0.111	-0.031	-0.098	-0.071	-0.025	-0.048	0.051	0.030	0.101	0.131	-0.080	0.101

Spearman's rank correlation coefficient.

*: q-value(False Discovery Rate (Benjamini & Hochberg)) < 0.05

Supplementary Table S4. Correlation of the amount of the change for one year between food and bacteria abundance (L_1-H_2).

		Soybeans and soybean foods	Green/yellow vegetables	Fruit	Fish/shellfish	Pickles	Mushrooms	Seaweeds	Green tea	Rice	Miso soup	Beef and pork	Coffee
Oral	Genus												
	Alloprevotella	-0.183	-0.010	0.176	0.166	0.103	0.018	0.256	-0.166	-0.268	0.150	0.263	-0.276
Gut	Phylum												
	Firmicutes	0.001	0.075	0.002	0.155	0.267	-0.210	0.270	-0.091	-0.106	-0.126	-0.033	-0.097
	Actinobacteria	0.082	0.382*	-0.258	-0.075	-0.113	0.167	-0.212	0.052	0.067	-0.040	0.260	0.078
	Class												
	Clostridia	-0.058	0.087	0.000	0.133	0.300	-0.236	0.262	-0.093	-0.130	-0.096	0.017	-0.072
	Negativicutes	0.148	-0.140	-0.021	-0.177	-0.294	0.038	-0.117	-0.088	-0.050	0.005	0.016	-0.080
	Actinobacteria	0.082	0.382*	-0.258	-0.075	-0.113	0.167	-0.212	0.052	0.067	-0.040	0.260	0.078
	Order												
	Clostridiales	-0.058	0.087	0.000	0.133	0.300	-0.236	0.262	-0.093	-0.130	-0.096	0.017	-0.072
	Selenomonadales	0.148	-0.140	-0.021	-0.177	-0.294	0.038	-0.117	-0.088	-0.050	0.005	0.016	-0.080
	Bifidobacteriales	0.115	.330*	-0.193	-0.128	-0.181	0.121	-0.297	0.072	0.043	-0.058	0.288	0.049
	Family												
	Ruminococcaceae	0.038	-0.268	0.083	-0.003	0.231	-0.083	-0.047	0.171	0.158	-0.079	-0.023	0.218
	Veillonellaceae	0.163	0.000	0.071	-0.172	-0.288	0.146	-0.116	0.040	0.053	-0.080	0.014	-0.085
	Bifidobacteriaceae	0.115	0.330*	-0.193	-0.128	-0.181	0.121	-0.297	0.072	0.043	-0.058	0.288	0.049
	Actinomycetaceae	-0.006	0.191	-0.132	0.302	0.018	0.205	0.131	0.034	-0.050	-0.006	0.044	0.048
	Genus												
	Faecalibacterium	-0.084	-0.142	-0.037	0.084	0.212	0.251	0.116	0.061	-0.013	-0.240	-0.023	0.043
	Gemmiger	0.024	-0.239	-0.197	0.143	0.031	0.046	-0.087	0.216	0.006	-0.183	-0.127	0.147
	Ruminococcus	-0.110	-0.121	0.361*	-0.203	0.163	-0.126	-0.226	0.124	0.079	0.008	0.165	0.082
	Lachnospiracea_incertae_sedis	-0.015	-0.153	0.265	-0.055	0.203	-0.176	0.037	0.193	0.069	-0.121	-0.161	0.021
	Bifidobacterium	0.115	0.330*	-0.193	-0.128	-0.181	0.121	-0.297	0.072	0.043	-0.058	0.288	0.049
	Actinomyces	-0.004	0.179	-0.120	0.299	0.017	0.202	0.144	0.039	-0.055	-0.001	0.053	0.046
	Parabacteroides	0.061	-0.106	0.092	-0.015	-0.167	0.133	0.143	-0.049	0.100	0.097	0.000	0.145

Spearman's rank correlation coefficient.

*: q-value(False Discovery Rate (Benjamini & Hochberg)) < 0.05

Supplementary Table S5. Correlation of the amount of the change for one year between food and bacteria abundance (H_1-H_2).

		Soybeans and soybean foods	Green/yellow vegetables	Fruit	Fish/shellfish	Pickles	Mushrooms	Seaweeds	Green tea	Rice	Miso soup	Beef and pork	Coffee
Oral	Genus												
	Alloprevotella	0.013	-0.032	-0.023	0.007	0.033	0.019	-0.054	0.130	0.046	-0.011	0.123	0.102
Gut	Phylum												
	Firmicutes	-0.017	-0.086	-0.112	0.025	-0.036	0.011	-0.185*	0.031	-0.031	-0.101	0.032	0.106
	Actinobacteria	-0.006	-0.120	0.089	0.059	-0.029	-0.023	0.064	0.033	-0.031	0.008	-0.003	-0.005
	Class												
	Clostridia	-0.004	-0.091	-0.157	0.048	-0.079	0.027	-0.170	0.018	0.014	-0.024	-0.015	0.092
	Negativicutes	0.026	0.194*	0.095	-0.033	0.074	-0.036	-0.007	-0.106	-0.033	-0.154	0.066	-0.083
	Actinobacteria	-0.006	-0.120	0.089	0.059	-0.029	-0.023	0.064	0.033	-0.031	0.008	-0.003	-0.005
	Order												
	Clostridiales	-0.004	-0.091	-0.157	0.048	-0.078	0.027	-0.170	0.018	0.014	-0.024	-0.016	0.093
	Selenomonadales	0.026	0.194*	0.095	-0.033	0.074	-0.036	-0.007	-0.106	-0.033	-0.154	0.066	-0.083
	Bifidobacteriales	-0.047	-0.066	0.034	0.094	-0.027	-0.047	0.111	-0.012	-0.049	0.069	0.006	-0.031
	Family												
	Ruminococcaceae	-0.018	-0.021	-0.272**	-0.037	-0.228**	-0.122	-0.126	0.019	0.079	0.047	0.112	0.081
	Veillonellaceae	-0.022	0.097	0.109	-0.081	0.067	-0.038	0.075	-0.043	0.004	-0.168	0.026	0.001
	Bifidobacteriaceae	-0.047	-0.066	0.034	0.094	-0.027	-0.047	0.111	-0.012	-0.049	0.069	0.006	-0.031
	Actinomycetaceae	0.040	-0.132	0.132	-0.069	0.067	-0.040	.180*	-0.077	-0.107	-0.150	0.027	0.042
	Genus												
	Faecalibacterium	0.050	-0.021	-0.105	-0.057	-0.075	-0.076	0.017	-0.001	0.023	-0.051	0.052	0.077
	Gemmiger	-0.233**	0.067	-0.160	0.027	0.160	-0.042	-0.122	0.017	-0.024	-0.007	0.097	0.066
	Ruminococcus	-0.008	-0.099	-0.125	-0.072	-0.206*	-0.104	-0.119	-0.059	0.183*	0.134	0.019	0.142
	Lachnospiracea_incertae_sedis	-0.098	0.043	0.019	0.143	0.076	-0.003	0.085	-0.043	0.018	0.090	0.025	-0.024
	Bifidobacterium	-0.047	-0.067	0.034	0.095	-0.029	-0.047	0.110	-0.013	-0.050	0.068	0.006	-0.030
	Actinomyces	0.022	-0.102	0.140	-0.046	0.058	-0.024	0.205*	-0.096	-0.123	-0.150	0.030	0.035
	Parabacteroides	0.040	0.237**	0.023	-0.060	0.069	0.073	-0.071	0.086	0.016	-0.142	0.058	-0.017

Spearman's rank correlation coefficient.

*: q-value(False Discovery Rate (Benjamini & Hochberg)) < 0.05, **: q-value(False Discovery Rate (Benjamini & Hochberg)) < 0.01

Supplementary Figure S1. Comparison of the diversity of oral microbiota among the four groups.

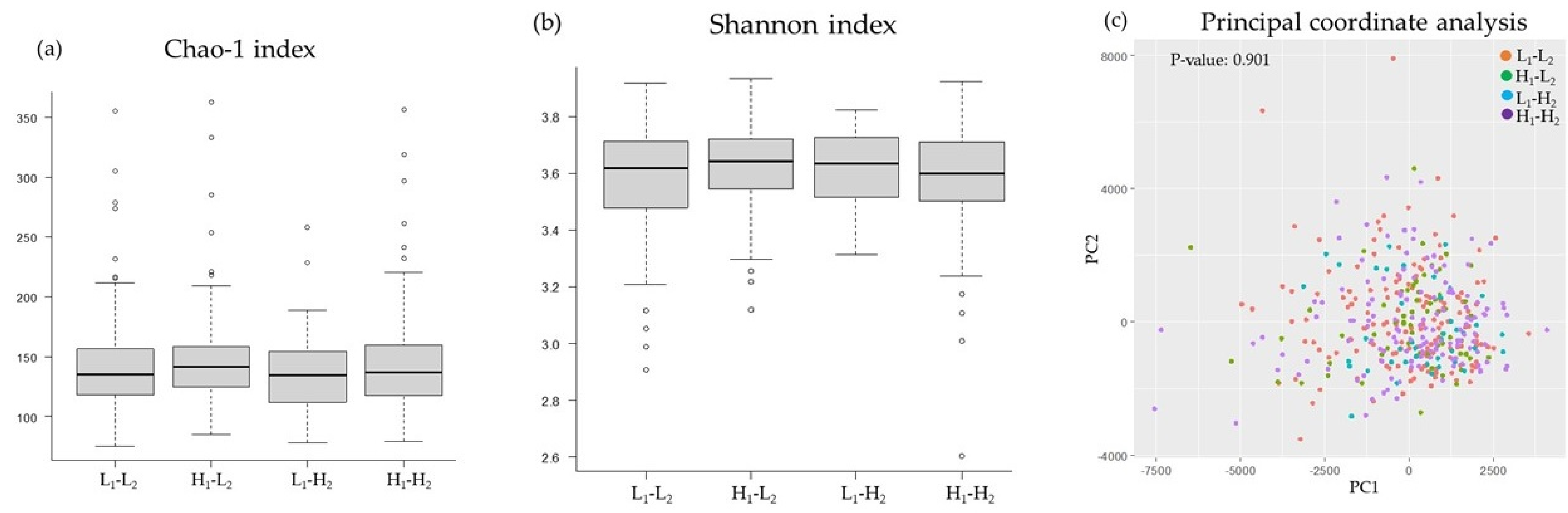

L1-L2: mJDI12 ≤ 6 in 2017 and 2018; H1-L2: mJDI12 ≥ 7 in 2017 and ≤ 6 in 2018; L1-H2: mJDI12 ≤ 6 in 2017 and mJDI12 ≥ 7 in 2018; and H1-H2: mJDI12 ≥ 7 in 2017 and 2018.

Supplementary Figure S2. Comparison of the diversity of gut microbiota among the four groups.

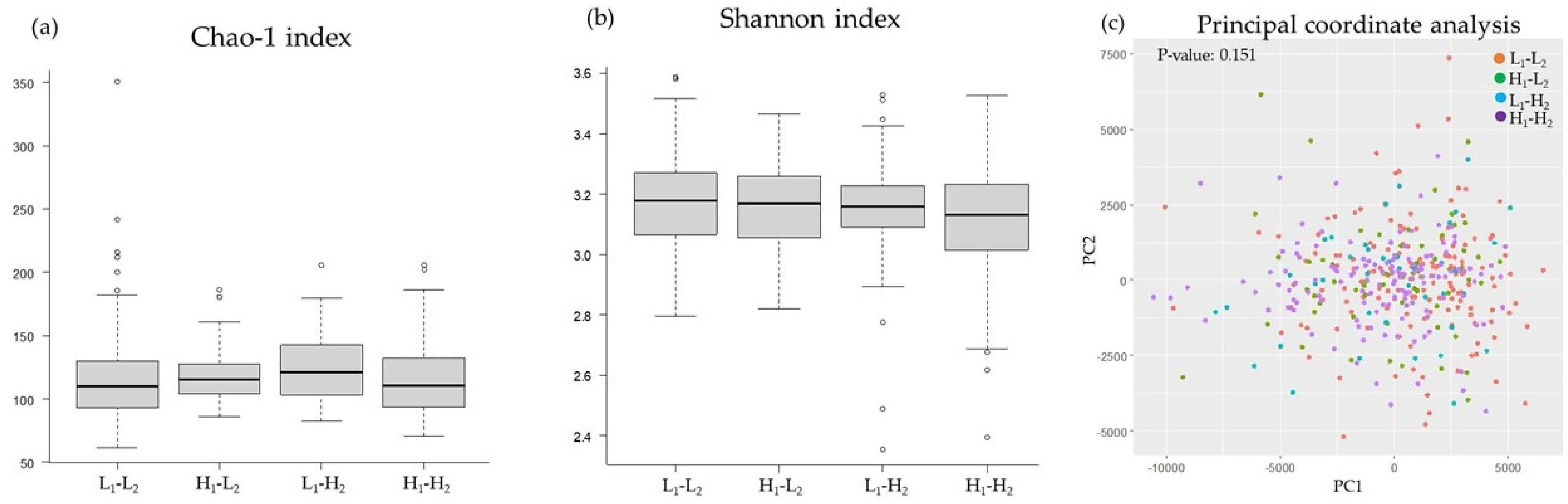

L1-L2: mJDI12 ≤ 6 in 2017 and 2018; H1-L2: mJDI12 ≥ 7 in 2017 and ≤ 6 in 2018; L1-H2: mJDI12 ≤ 6 in 2017 and mJDI12 ≥ 7 in 2018; and H1-H2: mJDI12 ≥ 7 in 2017 and 2018.
